# Origin of high strength, low modulus superelasticity in nanowire-shape memory alloy composites

**DOI:** 10.1038/srep46360

**Published:** 2017-04-12

**Authors:** Xudong Zhang, Hongxiang Zong, Lishan Cui, Xueling Fan, Xiangdong Ding, Jun Sun

**Affiliations:** 1 State Key Laboratory for Mechanical Behavior of Materials, Xi’an Jiaotong University, Xi’an 710049, China; 2 State Key Laboratory of Heavy Oil Processing, China University of Petroleum, Beijing, 102249, China; 3 State Key Laboratory for Strength and Vibration of Mechanical Structures, School of Aerospace, Xi’an Jiaotong University, Xi’an, 710049, China

## Abstract

An open question is the underlying mechanisms for a recent discovered nanocomposite, which composed of shape memory alloy (SMA) matrix with embedded metallic nanowires (NWs), demonstrating novel mechanical properties, such as large quasi-linear elastic strain, low Young’s modulus and high yield strength. We use finite element simulations to investigate the interplay between the superelasticity of SMA matrix and the elastic-plastic deformation of embedded NWs. Our results show that stress transfer plays a dominated role in determining the quasi-linear behavior of the nanocomposite. The corresponding microstructure evolution indicate that the transfer is due to the coupling between plastic deformation within the NWs and martensitic transformation in the matrix, i.e., the martensitic transformation of the SMA matrix promotes local plastic deformation nearby, and the high plastic strain region of NWs retains considerable martensite in the surrounding SMA matrix, thus facilitating continues martensitic transformation in subsequent loading. Based on these findings, we propose a general criterion for achieving quasi-linear elasticity.

A composite, combining together dissimilar materials, always gives rise to exciting properties superior to the properties of the individual components^1^. However, it is still challenge to sustain the high elastic strains and high strengths of metallic nanomaterials in their bulk composites^2^,^3^,^4^,^5^,^6^. Recently, Hao *et al*.^7^ used a unique composite system consisting of NWs and martensitic phase-transforming matrix, which named NWs *in situ* composite with SMA (NICSMA), and successfully achieved ultra-large elastic strains, low stiffness and high strength in solids at kilogram scale, which has great potential for applications in dental braces, cardiac pacemakers, implantable devices, and flexible medical instruments^8^,^9^. The approach also provide the possibility for designing phase transforming metal contained composites with desirable property combinations.

For a composite, the mechanical response to an external load is related not only to the inherent attributes of each of the components, but to the coupling between them as well^10^,^11^,^12^,^13^,^14^. The same is true for second-phase reinforced SMA composites, in which one component possesses intrinsic ultrahigh elastic strain limits and conventional elastic–plastic behavior^15^,^16^,^17^,^18^,^19^, and the other shows a superelasticity, which is mediated by a first order phase transformation^20^. Previous experimental works^21^,^22^,^23^,^24^,^25^ have indicated that the present of stress-induced martensitic transformation (SIMT) leads to effective load transfer between SMA matrix and nano-reinforcements. We take the Nb NWs strengthened NiTi SMA (a type of NICSMA) for example, the high energy x-ray diffraction and high resolution transmission electron microscopy experimental results show that the discrete occurrence of B2-B19′ martensite phase can impose a discrete local strain field on NWs at the site of the transformation region, thus making the Nb NWs experience a completely different plastic deformation behaviors^14^,^24^,^26^. To enhance the coupling between them, a mechanical pretreatment with several tensile strain loading cycles is always needed^7^,^14^,^24^,^27^. However, less is known about the connect between the microscopic coupling effect and the subsequent large quasi-linear elasticity.

In this work, we investigate the interplay between plastic deformation and phase transformation during the mechanical pretreatment, aimed at revealing the underlying determinants of the quasi-linear behavior of the SMA-NWs composite. We use the finite element method to simulate the mechanical response of the SMA-NWs composite during the pretreatment and subsequent loading cycles. Then, we establish a congruent relationship among stress transfer, volume fraction and aspect ratio of NWs as well as the value of pretreatment strain. Finally, we provide a guideline for the achievement of large quasi-linear elastic behaviors.

Here we first validate our simulation with previous synchrotron experiment. The NICSMA composite can be prepared experimentally by forging, wire-drawing, and annealing in macro-scale, e.g., macroscopic wires with 0.3~1.0 mm in diameters, and at kilogram scale^7^,^14^. To capture the typical microstructures (Fig. 1(a))^14^, the three-dimensional representative volume element approach is used, as shown in Fig. 1 (b) and (c). The interfaces between the SMA matrix and NWs in our finite element simulation are assumed as perfect bonding, as previous experiments showed that the microstructure of the composite consists of well dispersed and aligned Nb NWs (a mean diameter of 60 nm) embedded in the NiTi matrix with well-bonded interfaces, and there is no obvious debonding during deformation^7^,^14^.

Figure 2 shows the comparisons between experimental and simulated results. The volume fraction of NWs *V*_*N*_ = 25% is the same as that of the NICSMA^7^. The aspect ratio *L*/*d* = 20 is selected. Before benchmarking the quasi-linear superelastic behavior, a similar mechanical pretreatment with tensile strain cycle of 9.5% along the wire axial direction is applied^7^. Figure 2(a) shows the evolution of strain for the NWs and SMA matrix during the pretreatment. The strain of each component in our simulation is calculated according to the relationship between phase stress and lattice strain proposed by refs 21, 22, 23, 25 and 28. Figure 2(b) compares the cyclic tensile stress-strain curves of the SMA-NWs composite after the mechanical pre-treatment. Our simulation results (red lines) are approached to the experimental ones (blue lines). In addition, the macro-strain dependent average volume fraction of the martensite phase in our model (Fig. 2(c)) also shows a similar varying trend with that obtained from the relative intensity of the B19′-NiTi (001) diffraction peak (Fig. 2(d))^7^. All these results indicate that our FEM calculation predicts the mechanical response in excellent agreement with the experiments, which validates the implemented FEM model. We note that Fig. 2(a) reveals inconsistent unloading curves of NiTi matrix in the calculated (red curve) and experimental results (blue curve). This is because the simulated curve describes the mixed state of austenite-martensite phases, while the experimental result just present the B2 austenite phase estimated from the diffraction peaks of the B2-NiTi (211)^7^.

## Results

### Macro strain-stress curves before and after mechanical pretreatment

Figure 3(a) shows the stress–strain curves of the finite element model with the *V*_*N*_ = 25%, *L*/*d* = 20. The black and blue curves refer to the pretreatment (a tensile strain cycle of 7.5%) and the subsequent tensile cycle, respectively. During the pretreatment, the composite undergoes two-stage yielding, namely, the stress-induced martensitic transformation of SMA matrix and the plastic deformation of NWs. The corresponding microstructure evolution is demonstrated in Fig. 3(b). The red and purple correspond to mixed austenite–martensite (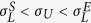) and pure martensite (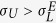) phases, respectively, where σ_*U*_ is equivalent stress (Mises stress) of the SMA matrix. The Mises stress σ_*U*_ below 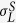 represents pure austenite. Upon loading, both the SMA matrix and NWs are deformed elastically. When the tensile strain approaches point A (Fig. 3(a)), the SIMT begins with the formation of the B19′ phase from both end regions of NWs (Fig. 3(b), A). Under further loading (Fig. 3(a), A to B), the SMA matrix is gradually transformed to a metastable microstructure mixed with austenite and martensite (Fig. 3(b), A to B, red region). This mixture of austenite and martensite keeps until point D (Fig. 3(a)). Pure martensite phase microstructure starts to form near the both ends of NWs at point D (Fig. 3(b)) and grow towards the interior of SMA matrix (Fig. 3(b), E). Upon unloading, a macro-residual strain about 1.3% is retained after pretreatment (Fig. 3(a), F). It is important to note that most martensite transforms back to parent phase, while some regions still retain a certain number of martensites due to the hindrance of plastic deformation within the NWs, as shown in Fig. 4(b).

The interaction between phase transformation in the SMA matrix and plastic deformation in the NWs during pretreatment is then studied. Figure 3(c) compares the corresponding volume fraction of martensitic phase of SMA matrix and equivalent plastic strain of NWs. The upper panel of Fig. 3(c) demonstrates the evolution of equivalent plastic strain within the NWs during the tensile loading of pretreatment, while the lower panel shows the corresponding volume fraction of martensite within the SMA matrix. In general, both the amount of plastic deformation and volume fraction of martensite increase with the macro-strain. However, it should be note that the plastic deformation of NWs prefers to be triggered and accumulated from the place where the martensitic transformation has occurred within the SMA matrix, as shown in the regions where the arrows point in Fig. 3(c) (The points C, D and E corresponding to Fig. 3(a)). The reason for this is that the elastic strain limit of the NWs is not large enough to match with the transformation strain of the SMA matrix, and the NWs are forced to deform plastically during the lattice distortion of martensitic transformation of the SMA matrix^24^,^29^.

Interestingly, a nearly linear superelasticity with narrow hysteresis is achieved in the subsequent loading cycle (Fig. 3(a), blue curve). The composite possesses a combination of high strength (>1.6 GPa), large pseudoelastic recovery strain (>6%), low Young’s modulus (25.6 GPa). To unveiling the contribution of each component on the quasi-linear superelasticity, we investigate the evolution of strain for NWs and SMA during the subsequent loading cycle, as shown in Fig. 4(a). Upon removal of the pretreatment load, the plastically deformed NWs hindered the recovery of the reverse transformation, which forces the SMA matrix to sustain a large residual tensile strain of ~1% (Fig. 4(a), H). At the same time, the NWs undergo an elastic compressive strain of ~1.4% (Fig. 4(a), G) due to the drag of the SMA matrix. The interaction between plastically deformed NWs and reverse phase transformation after pretreatment is shown in Fig. 4(b) and (c). Different from the loading process, after unloading, the area with high equivalent plastic strain value of NWs (Fig. 4 (c1)) is always accompanied by higher residual stress (Fig. 4(c2)) and larger volume faction of residual martensite (Fig. 4(c3)) in the surrounding SMA matrix. In short, local SIMT can promote the plasticity of surrounding NWs and the plastically deformed NWs subsequently hinder the reverse phase transformation. We thus emphasize the importance of pretreatment process in the resultant coupling we discussed above.

During the subsequent loading process, the pre-existing elastic compressive strain and an elastic tensile strain of 4.2% attribute to an ultra-wide elastic strain of the NWs. Accordingly, the strain-stress curve of the SMA matrix is confined to a slim hysteresis loop as shown in Fig. 4(a). We also find that the macro strain required to induce martensitic transformation has decreased from ~1.5% (Fig. 3(a), A) to ~0.25% (Fig. 4(a), I) with assistance of residual martensite that is generated during the mechanical pre-treatment. Subsequently, as Fig. 4(d) shows, we select six snapshots ((d1)–(d6) between I and J in Fig. 4(a)) to illustrate the microstructure evolution in subsequent loading process. Upon the subsequent loading, the martensitic transformation is initiated either from the areas with high residual stress (black arrows point in Fig. 4(d)) or from the growth of the pre-existing martensite nucleus (gray arrows point in Fig. 4(d)). Further tensile loading leads the martensite to grow in a more continuous way. As indicated by the arrows in Fig. 4(c) and (d), the volume fraction of martensite first increases from the location which surrounds high equivalent plastic strain regions of NWs. Then, the martensite region propagates smoothly toward other region of SMA matrix (from (d1) to (d6) in Fig. 4(d)), forming a spatially inhomogeneous pattern with different volume faction of martensite. This is quite different from that of the pretreatment process in which the martensitic transformation within SMA matrix occurs discontinuously (Fig. 3(b)). Thus, we can conclude that the coupling effect of the SIMT and plasticity is conducive to continuous phase transformation and leads to a quasi-linear superelasticity of the NICSMA. Note that this also implies that if the components generate a weak coupling during pretreatment, e.g., the NWs with small aspect ratio or volume fraction, or the composites undergo an insufficient pre-strain, the subsequent phase transformation within the matrix will become discontinuously, and show classic superelasticity.

### Effects of microstructure morphology and pretreatment strain on mechanical behavior

It is well-known that the mechanical property of metal matrix composite can be tuned by the microstructure morphology, e.g., shape and volume fraction of reinforcements. In our case, pre-strain is another critical factor because a suitable pretreatment is required to introduce the coupling effect between the plastic deformation of the reinforcements and the phase transformation of SMA matrix.

Figure 5(a) shows the stress–strain curves of the pretreated composites with volume faction of 25%, pre-strain of 7.5% and aspect ratio ranging from 2 to 30. The hysteresis loop decreases with the aspect ratio of NWs. When the aspect ratio *L*/*d* below 20, the curve is a classical nonlinear superelasticity associated with discontinuous SIMT. While *L*/*d* exceed 20 the composite exhibits a quasi-linear superelasticity, the hysteresis loops of which do not change any more. Our finding is similar to previous study on the tensile strength of carbon fibers reinforced magnesium matrix composites^30^. Therefore, in finite element analysis, given the trade-off relationship between efficiency and precision, the mechanical response of the NWs-SMA composite (when *L*/*d* > 20) could be represented by the model with NWs aspect ratio *L*/*d* = 20. We then study the influence of volume fraction of NWs, pre-strain on the characteristics of strain-stress curves, as shown in Fig. 5(b) and (c). With the increase of *V*_*N*_ (from 5% to 30%) or pre-strain (from 4.5% to 10.5%), both of yield strength and Young’s modulus increase, and a quasi-linear superelasticity is achieved. There also exists a critical value for *V*_*N*_ and pre-strain above which the shape of strain-stress curve keeps unchanged.

## Discussion

### A modified rule of mixture for NICSMA

For the composite after the pretreatment, the residual internal stresses between the NWs and matrix is created due to the mismatch in recoverable strains of the SMA matrix and NWs^14^,^24^. As the aforementioned results show, these residual stresses within the SMA matrix can affect the mechanical response of the composite by affecting the evolution of martensite phase transformation in subsequent loading cycles^7^,^14^. Therefore, we need to take residual stresses into the rule of mixture. After the pretreatment cycle, the average stress in each component of the composite consists of pure tension stresses 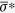 and residual stresses 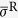. Thus, the yield strength of NICSMA can be modified as


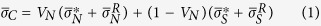


where *V*_*N*_ is the volume fraction of NWs, 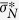 and 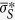 are the stresses due to pure tension in the NWs and SMA matrix respectively, and 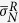, 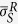 are the averaged residual stresses in the NWs and SMA matrix, respectively. As the residual stress should be in the elastic region, the averaged residual stresses in the NWs and SMA matrix can be written as 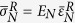 and 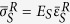, where *E*_*N*_, *E*_*S*_, 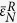 and 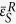 are the elastic modulus and residual strain of the NWs and the SMA matrix respectively.

In addition, the stress in NWs due to tension of the pure elastic strain can be expressed as 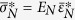, 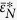 is the pure tension strain of the NWs. During loading process, the SMA matrix will undergo an initial elastic deformation followed by a SIMT transition^20^,^31^, thus, the stress in SMA matrix due to tension can be written as


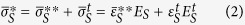


here 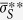 is the stress of SMA matrix, 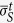 is the stress of phase transformation, 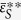 the elastic strain of SMA matrix, 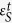 the strain of martensite phase transformation, and 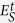 the elastic modulus during phase transformation. Finally, we get the yield strength of the composite as


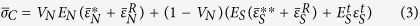


Similarly, the elastic modulus of the composite is


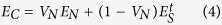


According to equations (3, 4), the calculated yield stress and elastic modulus in our case are 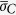 = 1.65 GPa and 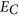 = 25.5 GPa, respectively. Both values agree very well with previous experimental results (yield strength and elastic modulus of NICSMA are, respectively, 1.65 GPa and 25.8 GPa)^7^.

### Stress transfer effect

As discussed above, the interaction between the NWs and the SMA matrix plays an important role in the quasi-linear superelasticity. In the SMA-NWs nano-composite, the NWs contribute to the overall load bearing capacity of the composite, whereas the SMA matrix leads to the effective load transfer between the components. To qualify this interaction, we introduce a parameter, i.e., stress transfer factor,


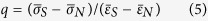


where 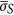, 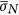, 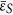 and 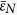 are the axis stresses and strains of the SMA matrix and the NWs, respectively. The definition of q is shown in Fig. 6(a). A larger absolute value of *q* (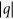) indicates more stress is transferred from NWs to SMA matrix. Therefore, it is possible to reveal the transformation behavior of the composite by the variation of 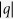 and its second order derivative 
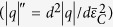
32,33,34,35.

Figure 6(b) and (c) shows the correlations among 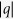, 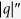 and macro-stress for the composite during the pretreatment. We find that 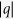 keeps constant when the macro-strain is below point A (Fig. 6(b)), indicating a pure elastic deformation process. This value is much greater than that of dual-phase steels as well as other well-known fiber reinforced metal matrix composites^32^,^33^,^34^,^35^,^36^. We believe it is due to the unique properties of the SMA matrix. The 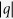 value then decreases sharply with the applied strain as a result of the martensitic transformation of SMA matrix. Upon further loading, 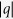 decreases much slowly when the NWs start to plastic deformation (point C).

The deformation stages of second-phase reinforced SMA nanocomposite are distinguished by stress-strain curves as well as the lattice strain obtained from high energy x-ray diffraction results^21^,^23^,^25^,^37^,^38^. Here, we show that the deformation stages of the nanocomposite are clearly reflected in the 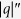 curve, where different stages are well separated by its peaks (Fig. 6(c), A, B and C). The first distinct extremum (point A) stands for the initiation of SIMT. It means that stage I (before point A) is the elastic regime, in which both the NWs and the SMA matrix deformed elastically. After point B (the second extremum), the entire SMA matrix is full of the mixed austenite–martensite, and transforms to mature martensite variants. We defined this region as stage II (that is between A and C). The extremum of 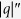 at point C is caused by the occurrence of local plastic deformation of NWs, leading to the starting of stage III.

We have shown that the residual stresses of SMA matrix are created due to the interaction between the plastically deformed NWs and the reverse transformation of SMA matrix. We accordingly infer that there is a possible connection between stress transfer and residual stress. Figure 7(a) shows the calculated stress transfer factor 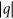 (blue points) and mean SMA matrix residual stress (black points) of a pretreated (pre-strain = 7.5%) composites with NWs volume fraction *V*_*N*_ = 25%, aspect ratio ranging from 2 to 30. We find that both the stress transfer factor and residual stress first increase with the aspect ratio, and then keep a constant value when the aspect ratios are larger than 20. Figure 7(b) shows the calculated results of the composites with NWs volume fraction *V*_*N*_ = 25%, NWs aspect ratio *L*/*d* = 20, pre-strain ranging from 5.5% to 7.5%. Similarly, the stress transfer factor and residual stress increases with the pre-strain, but the growth slows when the pre-strain is over 7.5. The effects of aspect ratio and pre-strain on stress transfer resemble that on residual stress. However, we find that the effect of NWs volume fraction is opposite, as shown in Fig. 7(c), consistent with previous studies on other fiber reinforced metal matrix composite^32^,^33^. These studies also demonstrate that the coupling between NWs and SMA matrix can be tuned by the three primary parameters.

We then explore how the stress transfer after pretreatment affects the mechanical behavior in subsequent tensile cycle. We compared the 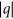 value with average residual stress of SMA matrix with a constant NWs volume fraction *V*_*N*_ = 25%, ranging from 5.5% to 9.5% and 2 to 30 in pre-strain and aspect ratio, respectively (Fig. 8(a) and (b)). Each of the results was divided into two areas, for the 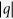 (Fig. 8(a)), the boundary is determined by the plateau level where the 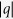 show almost constant with the increase of NWs aspect ratio continually, for residual stress of matrix (Fig. 8(b)), the boundary is determined by the final stress for the reverse phase transformation (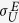). Obviously, both boundaries are coincident, which indicate that deformation behavior of the composite is essentially determined by the stress transfer effect. Therefore, stress transfer factor 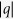 can be used as the criterion to achieve quasi-linear superelasticity.

### Guideline for achieving quasi-linear superelasticity

Based on the criterion of stress transfer factor, we provide a guideline for achieving quasi-linear superelasticity of the SMA-NWs composite. We take the SMA matrix composite with 25% volume faction of NWs for example. Figure 8(a) and (b) shows the phase diagram of it as a function of aspect ratio and pre-strain. Large linear superelasticity can be achieved in the blue region, which is above a dash curve where 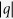 = 39 or residual stress = 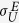. Below the dash curve (the white region), the mechanical response of the SMA-NWs composite is a normal superelasticity. Both Fig. 8(a) and (b) show that quasi-linear superelasticity only appears under the conditions that the nanocomposite experiences a considerable pre-strain and the embedded NWs have a relative large aspect ratio. Subsequently, we draw a 3D phase-diagram of the shape of superelasticity by considering these three parameters, i.e., volume faction of NWs, aspect ratio of NWs, and pre-strain. As shown in Fig. 8(c), only when the value of 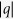 is above the 3D threshold surfaces, the nanocomposite can possess a large quasi-linear superelasticity, otherwise, the materials show a normal superelasticity. In addition, we find that the threshold of required global pre-strain and aspect ratio increase with the decrease of the NWs volume fraction and the required pre-strain increase with the reduction of the aspect ratio of embedded NWs. We believe that these mechanisms and guidelines should also apply to other SMA based nanocomposites.

In conclusion, our simulation results intuitively revealed the interactions between forward/reverse phase transformation and elastic-plastic deformation. The local product phase facilitates the accumulation of local plastic strain. Upon unloading, the plastically deformed NWs in turn hinder the reverse phase transformation, which causes retained more martensite phase, and the martensitic transformation initiate from these special areas in subsequent loading process. We find that the stress transfer determines the mechanical behavior of the composite. By controlling the stress transfer effect, we provided a scheme for tailoring desirable mechanical properties, which has the potential to design new SMAs matrix composites.

## Methods

### Finite element modelling

**Figure 1:**
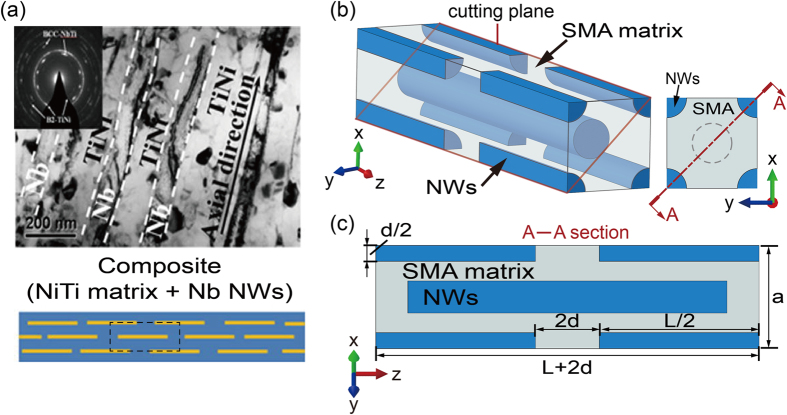
Typical microstructure and FE geometric model of the nanowire *in-situ* composite with SMA (NICSMA). (**a**) Microstructure of the TiNi-Nb *in-situ* nanocomposite obtained from experiment^14^. (**b**) 3D periodic unit cell structure of the NICSMA. (**c**) Sectional illustration of a longitudinal section of the staggered NWs embedded in SMA matrix.

**Figure 3:**
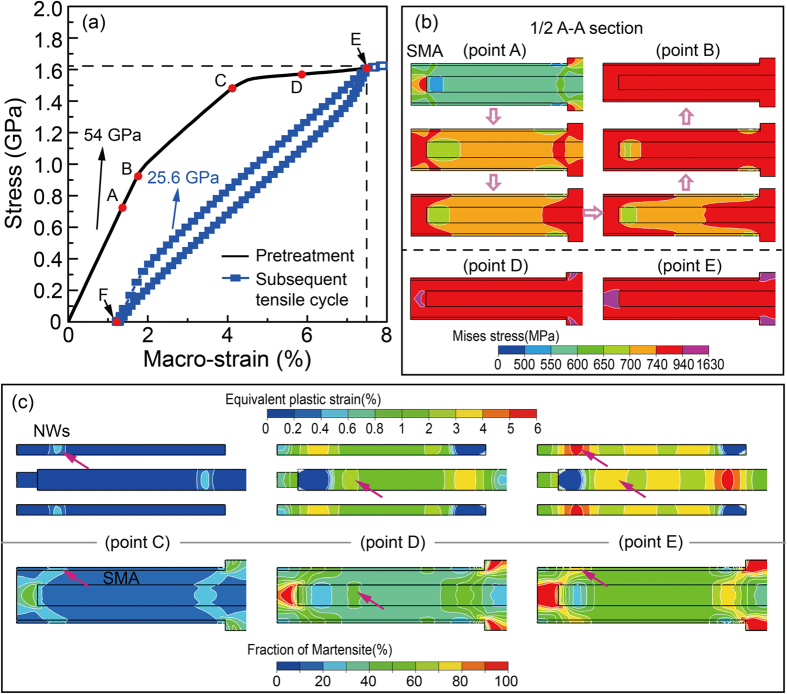
(**a**) Macro stress-strain curves of the composite during pretreatment and subsequent loading cycle. (**b**) Evolution of martensitic transformation during the pretreatment process; the red and purple correspond to mixed austenite–martensite and pure martensite
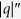
phases respectively (only show half of the section due to the symmetry). (**c**) Local martensite phase transformation within SMA facilitates the plasticity of NWs during the first loading process; the red arrows in upper panel point the area signifying the onset and accumulation of the plasticity of NWs, and in lower panel mean the areas containing more martensite phase. Points A-E are the corresponding locations in (**a**).

**Figure 4:**
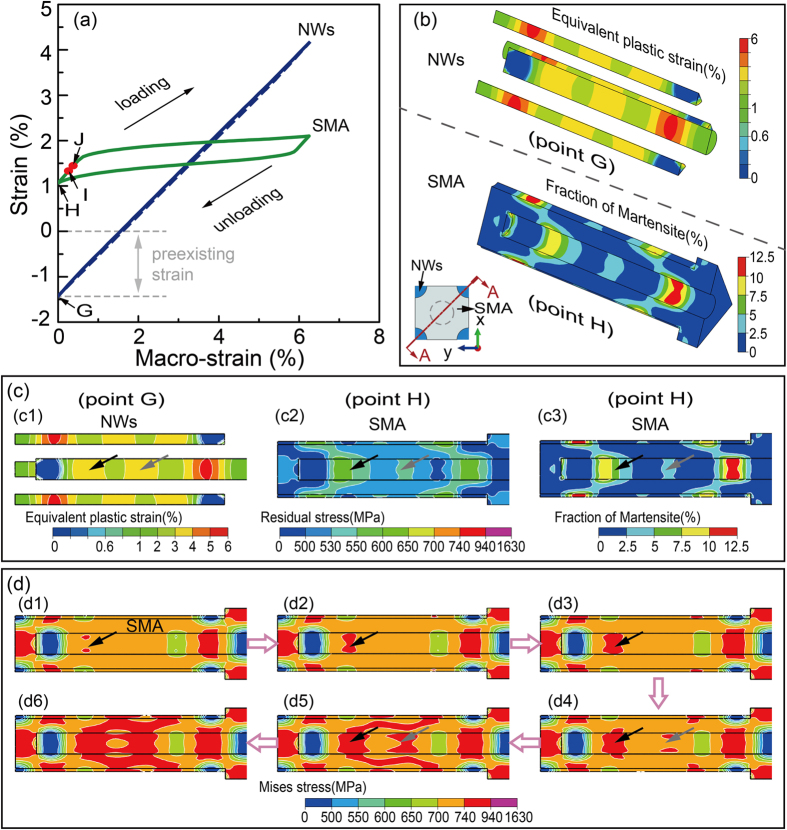
(**a**) Evolution of strain for NWs and SMA during the subsequent tensile cycle. (**b**) Sectional view showing the distribution of equivalent plastic strain of the NWs and residual martensite within the SMA matrix after pretreatment (half of the section only); Points G and H correspond to the states located in (**a**). (**c**) The plastically deformed NWs hindered the recovery of the reverse transformation of SMA matrix upon unloading; (c1) Equivalent plastic strain of the NWs; (c2) Residual stress within the SMA matrix; (c3) Volume fraction of retained martensite within the SMA matrix after unloading; the black and gray arrows point to the correspondences between plasticity of NWs and residual states of SMA matrix. (**d**) Evolution of martensite phase transformation in subsequent loading process; in (d1)–(d6), the arrows point to the emergence of martensite (between point I and J in (**a**)).

**Figure 5:**
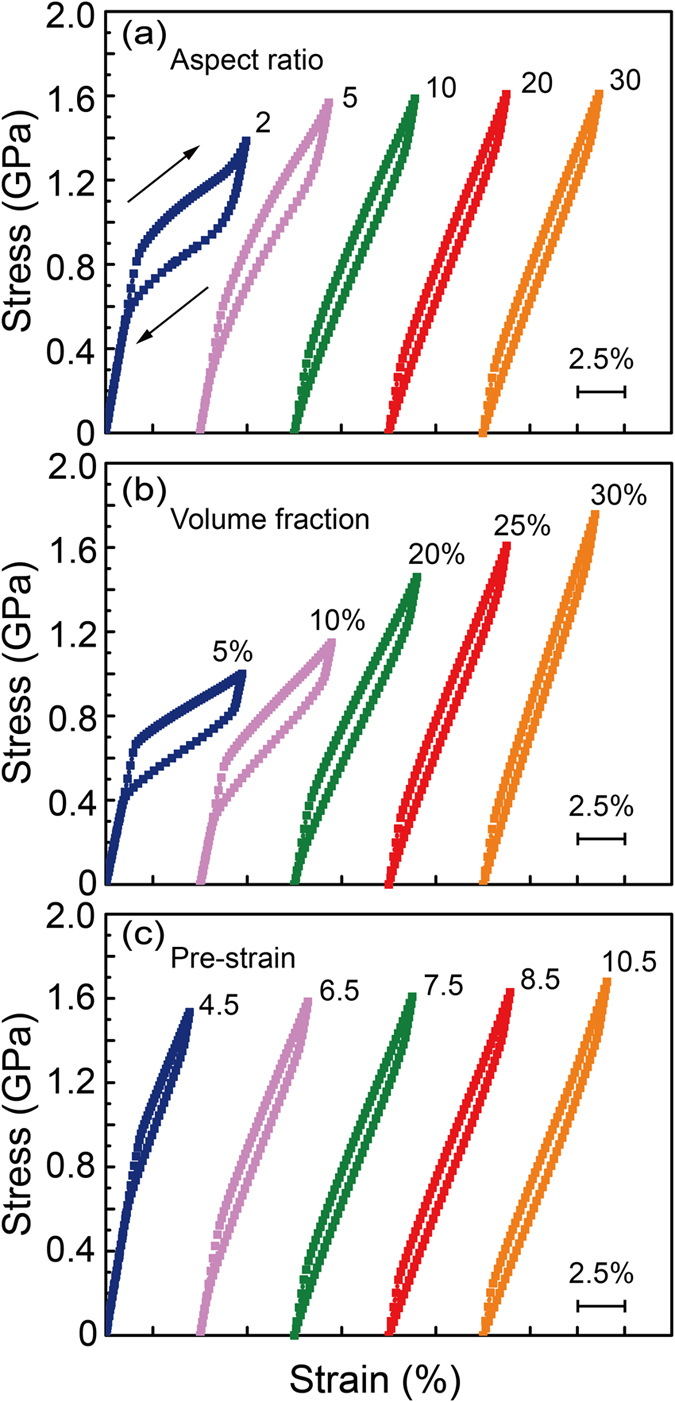
Effects of (**a**) aspect ratio of NWs, (**b**) volume fraction of NWs and (**c**) pretreatment strain on the stress-strain curves of the composites in the subsequent tensile cycle.

**Figure 6:**
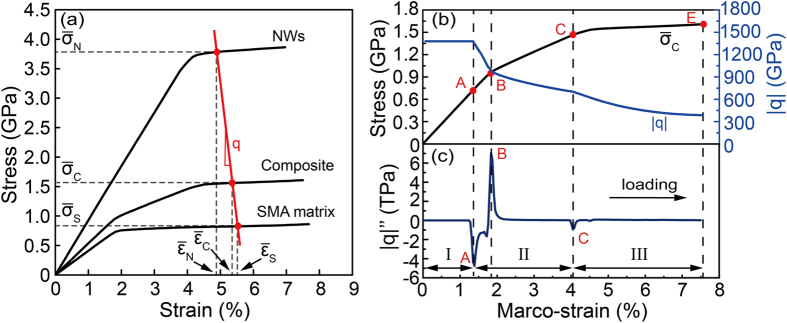
(**a**) Stress-strain curves of the components and composite; q is defined as the slope of the red line. (**b**) Stress transfer factor 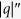 (blue line) and stress-strain curve of the composite (black line), indicating that the martensitic transformation of SMA matrix could result in the decrease in the stress transfer effect. (**c**) The corresponding curve of 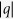. The changes of 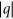 clearly distinguish the three deformation stages (indicated by I, II, III, respectively) of NICSMA during the loading process of the pretreatment.

**Figure 7:**
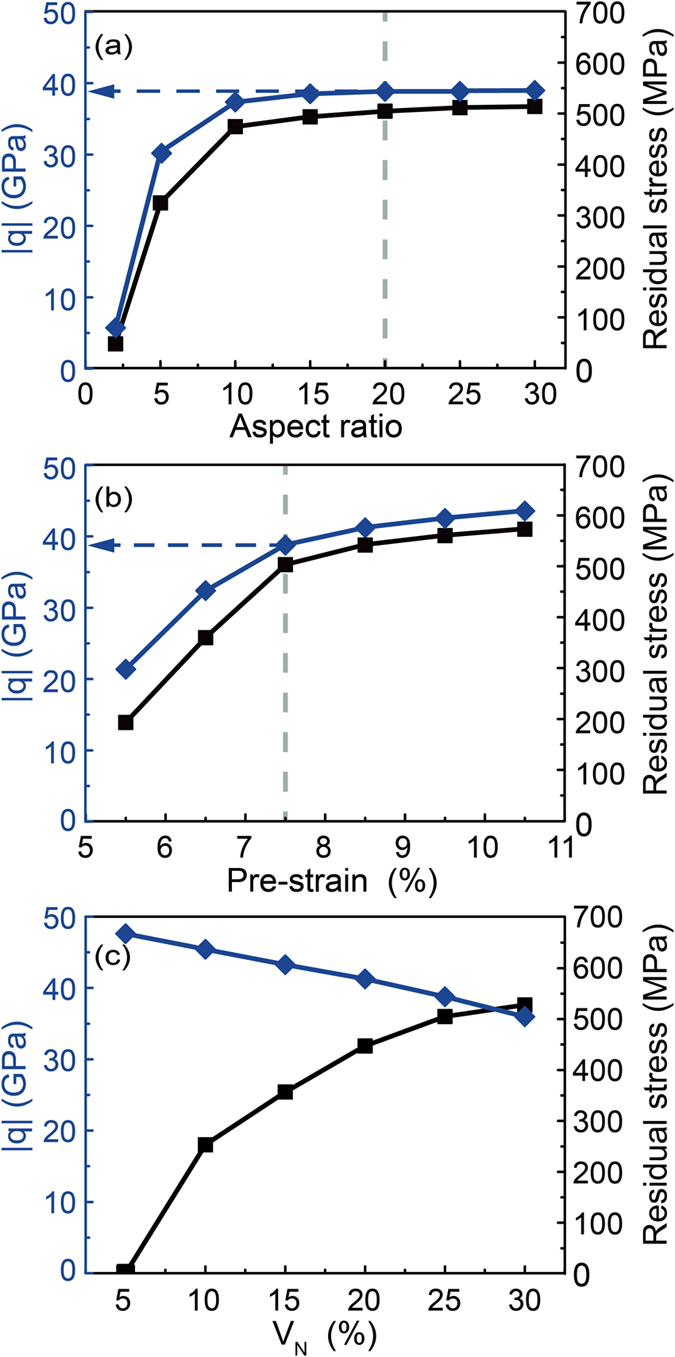
Comparisons of stress transfer factor 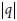 and mean residual stress of SMA matrix upon unloading as a function of (**a**) aspect ratio of NWs, (**b**) macro-pretreatment strain, and (**c**) volume fraction of NWs. The coupling effect between SIMT of SMA matrix and plasticity of NWs can be tailored by these three primary parameters.

**Figure 8:**
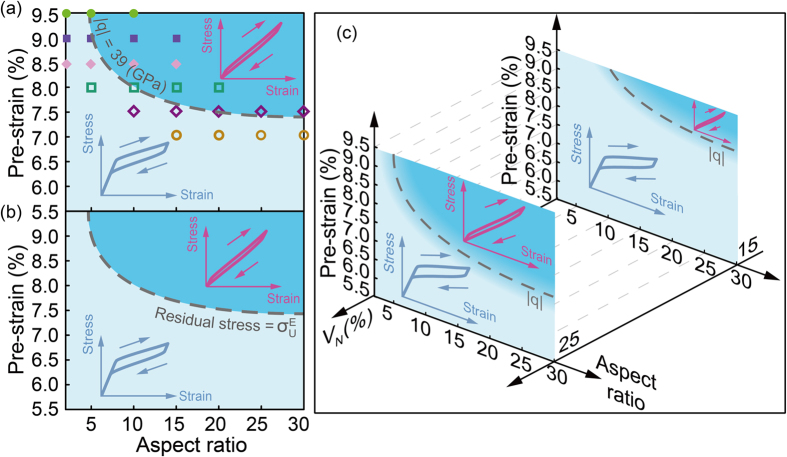
A general criterion for achieving quasi-linear elasticity of NICSMA. Large linear superelasticity can be achieved in the blue region. (**a**) Stress transfer factor 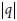 and (**b**) mean residual stress of SMA matrix as a function of aspect ratio and pre-strain (25% volume fraction of NWs). (**c**) Design guideline for large linear superelasticity of NICSMA. We select only two slices (15% and 25%) along the axis of volume fraction of NWs, *V*_*N*_.

As Fig. 1(c) shows, the volume fraction of NWs (*V*_*N*_) is determined by *a*, the diameter *d* of NWs is 60 nm, and several length *L* is selected to study the effect of aspect ratio (*L*/*d*) on the macroscopic mechanical response. Two uniaxial tensile loading cycles along the wire axial direction (Z axis, Fig. 1(b)) are simulated to reproduce the pretreatment process and the subsequent mechanical behavior. A periodic boundary condition is applied to avoid unrealistic effects caused by symmetric boundary conditions^39^,^40^. The volume average approach performed by a Python code is applied to compute the stress and strain fields and fraction of martensite within the composites^32^,^33^.

### Constitutive equation of SMA and NWs

In order to simulate the superelastic behavior related NiTi matrix, a constitutive model for nitinol^41^,^42^, which is proposed by Auricchio and Taylor, is employed^43^,^44^. In this model, the superelastic behavior consists of a purely linear elastic segment and a transformation platform. All the parameters are obtained by fitting the main features of the existing experimental results^7^. Accordingly, the major input parameters include the starting stress (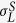 = 740 MPa) and final stress (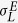 = 940 MPa) for the forward phase transformation, the starting stress (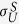 = 700 MPa) and final stress (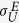 = 500 MPa) for the reverse phase transformation, the elastic modulus of parent phase (*E*_*A*_ = 41 GPa) and martensite (*E*_*M*_ = 41 GPa) and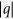 the transformation strain (ε^*L*^ = 0.1). The mechanical response of Nb NWs is described by an ideal elastic-plastic body with yield strength σ_*NS*_ of 4 GPa, elasticity modulus *E*_*N*_ of 93 GPa^45^, and Poisson’s ratio *ν*_*N*_ of 0.33.

## Additional Information

**How to cite this article**: Zhang, X. *et al*. Origin of high strength, low modulus superelasticity in nanowire-shape memory alloy composites. *Sci. Rep.* **7**, 46360; doi: 10.1038/srep46360 (2017).

**Publisher's note:** Springer Nature remains neutral with regard to jurisdictional claims in published maps and institutional affiliations.

## Figures and Tables

**Figure 2:**
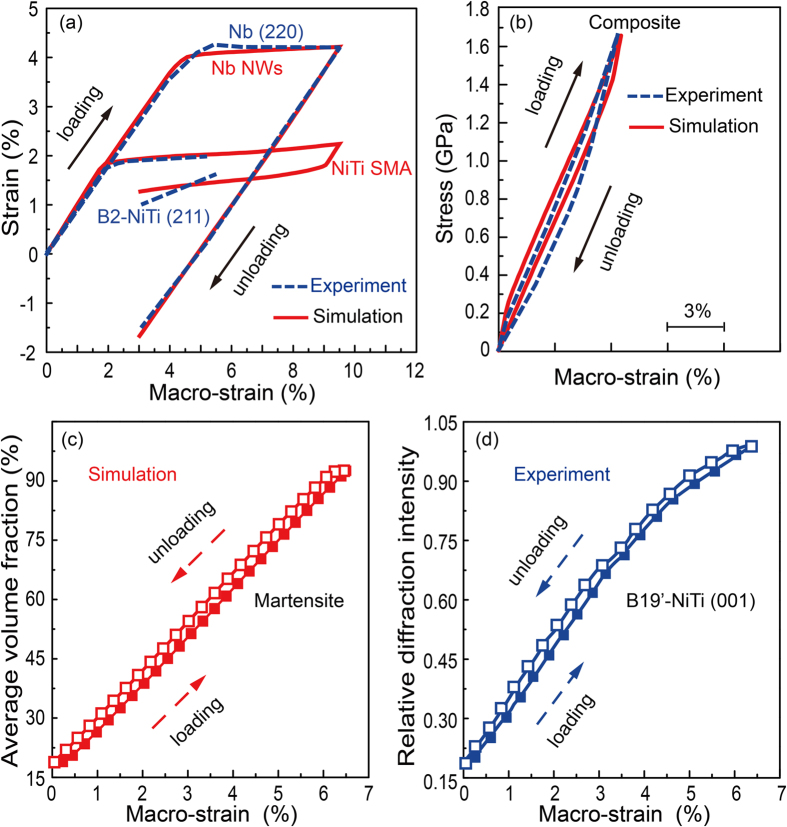
Comparisons between experimental and simulated results, showing the validity of the implemented simulation method. (**a**) Evolutions of strain for NWs and SMA matrix during the pretreatment. (**b**) Cyclic tensile stress–strain curves of the composite during the subsequent tensile cycle. (**c**) Evolution of the mean volume fraction of martensite phase during the subsequent tensile cycle in simulation. (**d**) Evolution of the relative intensity of the B19′ diffraction peak during the subsequent tensile cycle in experiemt^7^.
